# Frailty Predicting Health-Related Quality of Life Trajectories in Individuals with Sarcopenia in Liver Cirrhosis: Finding from BCAAS Study

**DOI:** 10.3390/jcm12165348

**Published:** 2023-08-17

**Authors:** Deepak Nathiya, Preeti Raj, Pratima Singh, Hemant Bareth, Arun Singh Tejavath, Supriya Suman, Balvir Singh Tomar, Ramesh Roop Rai

**Affiliations:** 1 Department of Pharmacy Practice, Institute of Pharmacy, Nims University, Jaipur 303121, Rajasthan, India; dnathiya@nimsuniversity.org (D.N.); rajpreetipp@gmail.com (P.R.); barethhemant@gmail.com (H.B.); supriyasingh3011@gmail.com (S.S.); chancellornims@gmail.com (B.S.T.); 2 School of Public Health, University of Alberta, Edmonton, AB T6G 2R3, Canada; 3 Department of Gastroenterology, National Institute of Medical Science, Nims University, Jaipur 303121, Rajasthan, India; tejarun0708@gmail.com (A.S.T.); rameshroop@gmail.com (R.R.R.); 4 Institute of Pediatric Gastroenterology and Hepatology, Nims University, Jaipur 303121, Rajasthan, India

**Keywords:** liver cirrhosis, frailty, sarcopenia, quality of life

## Abstract

The association between frailty and health-related quality of life (HRQoL) among Asian patients with liver cirrhosis and sarcopenia remains largely unexplored. To address this knowledge gap, we conducted a cross-sectional study involving individuals aged 32 to 69 years, all diagnosed with liver cirrhosis. The chronic liver disease questionnaire (CLDQ) was used to assess HR-QoL, the CLDQ score was used as an outcome to measure the factors related to HR-QoL, and the liver frailty index (LFI) was used to assess the frailty status. The association between the frailty status and the CLDQ summary scales was investigated using the correlation coefficient and multiple regression analyses. A total of 138 patients in the frail (*n* = 62) and non-frail (*n* = 76) groups with (alcohol: 97; viral: 24; autoimmune: 17; and cryptogenic: 12) were included in the study. Age, CTP score, and model for end-stage liver disease (MELD) sodium were significantly higher in the frail group. In the CLDQ domains, there was a significant difference between the frail and non-frail groups (*p* value = 0.001). In health-related quality-of-life summary measures, there was a strong negative correlation between frailty and the scores for activities, emotional function, and fatigue (*p* value = 0.001). When comparing frail to non-frail patients, these characteristics demonstrated significantly increased odds as indicated by their adjusted odds ratios: OR 3.339 (*p* value = 0.013), OR 3.998 (*p* value = 0.006), and OR 4.626 (*p* value = 0.002), respectively.

## 1. Introduction

Liver cirrhosis, a complex and multifaceted chronic liver disease characterized by progressive fibrosis and the replacement of functional hepatic parenchyma by nodular regeneration and scar tissue, has emerged as a significant global health concern due to its escalating prevalence and mortality rate [1,2]. Its progression can result in a variety of complications, including non-alcoholic fatty liver disease; portal hypertension; hepatocellular carcinoma; and, ultimately, liver failure.

Amid the numerous challenges posed by liver cirrhosis, frailty has been recognized as a prevalent and potent risk factor, capable of intensifying the disease’s adverse outcomes [3]. Frailty is a multifaceted syndrome involving the cumulative decline in various physiological systems, leading to vulnerability to stressors and adverse health outcomes. It is frequently delineated by sarcopenia, a debilitating decline in muscle mass, strength, and functionality that also includes various physiological, psychological, and socio-environmental aspects [3,4]. Chronic conditions, such as liver cirrhosis, not only predispose individuals to frailty but can also exacerbate its progression due to the imposed psychological stress, prolonged illness duration, and decreased physical activity [5].

The Indian subcontinent presents a unique context for understanding the interplay of liver cirrhosis, frailty, and health-related quality of life (HR-QoL). The region’s distinctive socio-cultural milieu, characterized by specific dietary practices, disparate healthcare accessibility, and prevalent comorbidities, influences the demographic and clinical profile of liver cirrhosis and frailty [6]. Despite this, the nexus between frailty and HR-QoL within this specific population has been inadequately investigated. HR-QoL is a multidimensional concept that captures an individual’s perceived physical, psychological, and social well-being, thereby offering a comprehensive reflection of their overall health status. It is particularly crucial in chronic diseases such as liver cirrhosis as it encapsulates the repercussions of the disease and its treatment on the patient’s everyday life [6,7]. Prior research has established a link between frailty and diminished HR-QoL across various chronic conditions, including liver diseases [8]. However, these investigations predominantly rely on generic assessment tools like the SF-36, which may fail to capture the unique clinical manifestations and challenges presented by liver cirrhosis [9,10]. Therefore, our study seeks to adopt a more disease-specific lens to assess HR-QoL, thus filling a critical gap in the existing literature.

In this study, we aim to provide a more nuanced perspective by using disease-specific assessment tools for HR-QoL. We intend to investigate the prevalence of frailty and the relationship between frailty status and HR-QoL trajectories in sarcopenic patients with liver cirrhosis from the Indian subcontinent. By forecasting HR-QoL trajectories based on frailty status, we hope to provide a dynamic understanding of the progression of HR-QoL in this specific population. Furthermore, this will foster an improved and patient-centric approach to the care of these individuals, thus promoting better health outcomes and enhancing their quality of life.

## 2. Materials and Methods

### 2.1. Study Population and Design

The current analysis is part of a comprehensive project, the BCAAS study [11]. As a cross-sectional and hospital-based examination, this research was carried out within the Department of Gastroenterology at the National Institute of Medical Sciences and Research, NIMS University Rajasthan, Jaipur, India from April 2019 to August 2020.

The research protocol strictly adhered to the 1975 Declaration of Helsinki principles and the World Association for Public Opinion Research (WAPOR) reporting guidelines, ensuring robust ethical integrity [12,13]. The institutional ethics committee granted approval for this study, as confirmed by IEC No NIMSUNI/IEC/217/22. All participants were briefed comprehensively about the study’s objectives, methodology, and potential implications. Subsequent to this, written informed consent was obtained from each participant, reinforcing their autonomy and their right to withdraw from the study at any stage without repercussions.

The study population comprised patients diagnosed with liver cirrhosis (LC), with the diagnosis confirmed by a combination of clinical, laboratory, and histopathological evidence. The severity of liver disease was ascertained by the Child–Turcotte–Pugh Score (CTP), with scores ≥7 and <12, corresponding to class B or C, serving as inclusion criteria [14]. Radiological and endoscopic evidence of portal hypertension further underscored the severity of their hepatic condition.

In line with the study’s focus on frailty in the context of liver cirrhosis, all participants were also diagnosed with sarcopenia, as defined by the European Working Group on Sarcopenia in Older People 2, 2018 (EWGSOP2) cut-off values [15,16].

However, patients with significant ascites or overt hepatic encephalopathy were excluded due to the potential confounding effect of these conditions on cognitive function and subsequent self-reporting during HR-QoL assessments.

### 2.2. Detailed Study Procedure

After enrollment, participants underwent a comprehensive clinical evaluation that started with an extensive medical history review. This focused on elements such as the duration and progression of liver disease, patterns of alcohol consumption, ongoing medication regimen, and co-existing diseases. Such data allowed for a broader understanding of the patient’s overall health status and potential factors influencing their liver condition.

This was followed by a detailed physical examination to identify clinical signs of chronic liver disease. A particular emphasis was placed on assessing the severity of sarcopenia, measured through the handgrip strength test. This tool is non-invasive, user-friendly, and offers a reliable measure of the muscle function in patients.

Concurrently, a comprehensive set of laboratory investigations was carried out. The full blood count provided information on the patient’s hematological status. Liver function tests, which include measurements of total bilirubin, aspartate transaminase (AST), alanine transaminase (ALT), and albumin levels, were conducted to assess hepatic functional capacity. A coagulation profile, encompassing prothrombin time (PT), the international normalized ratio (INR), and activated partial thromboplastin time (aPTT), was employed to identify coagulopathy, a common complication in chronic liver disease. Serum electrolytes and renal function tests (serum creatinine, urea) were analyzed to detect any signs of electrolyte imbalances and evaluate renal function, respectively.

In addition, the model for end-stage liver disease (MELD) and Child–Pugh scores were calculated using biochemical parameters (INR, creatinine, bilirubin, etc.), and clinical findings (encephalopathy, ascites) to obtain a more objective evaluation of liver disease severity.

Lastly, imaging modalities such as abdominal ultrasonography and endoscopy were utilized for the detailed visualization of liver architecture, the evaluation of portal hypertension, and the identification of potential sequelae of liver disease. These techniques allowed for a more in-depth understanding of the severity and progression of the liver disease, helping one to obtain more accurate patient stratification.

### 2.3. Health Status Measurement Tool

Two primary instruments were used for assessing health status among our study participants: the liver frailty index (LFI) and the chronic liver disease questionnaire (CLDQ).

#### 2.3.1. Liver Frailty Index

It is a validated measure used for assessing frailty in patients with liver disease. All enrolled patients underwent frailty testing conducted by trained personnel using the liver frailty index (LFI). The LFI quantitatively evaluates frailty using three key performance-based physical function assessments:

1. Grip strength: the average of three trials was measured using a hand dynamometer in the subject’s dominant hand.
2. Timed chair stands: the time taken for a subject to complete five chair stands with their arms folded across the chest was recorded.
3. Balance testing: the number of seconds a patient could maintain three postures (side-to-side, semi-tandem, and tandem) for a maximum of ten seconds each was recorded.

The LFI was calculated using the following formula incorporating these three assessments: LFI = (−0.330 × gender-adjusted grip strength) + (−2.529 × number of chairs stand per second) + (−0.040 × balance time) + 6.

Subjects were then categorized based on previously established LFI cutoffs: robust (LFI ≤ 3.2), prefrail (LFI between 3.2 and 4.4), and frail (LFI ≥ 4.5). However, given that our study’s primary focus is to distinguish between frail and those who are not, subjects were subsequently re-categorized into two broader categories: non-frail (incorporating both the robust and prefrail groups) and frail [17].

#### 2.3.2. Chronic Liver Disease Questionnaire (CLDQ)

This questionnaire served as a disease-specific instrument to measure longitudinal changes in health status among individuals with chronic liver disease in our study. This tool consists of 29 items distributed across six domains: “fatigue”, “emotional function”, “worry”, “activity”, “abdominal symptoms”, and “systemic symptoms”. Each item utilizes a seven-point Likert scale, with 1 indicating the absence of a symptom or feeling and 7 indicating the highest frequency or intensity of the symptom or feeling.

The administration of the CLDQ was carried out by trained research personnel. Participants were provided clear instructions, and queries were addressed to ensure accurate and reliable responses. The results were analyzed both at the domain and overall level. The scores for each domain were computed as the mean score of items within the domain, while the overall CLDQ score was computed as the mean of all 29 items. A higher score indicated a better health-related quality of life.

As for the reliability and validity of CLDQ, it has been widely acknowledged as a robust tool in various cultural contexts, as well as correlating strongly with disease severity. In our study, the internal consistency of the questionnaire was confirmed by a high Cronbach’s alpha across its domains, with the total questionnaire demonstrating an alpha of 0.93. The individual domain alphas were as follows: abdominal symptoms 0.94, activity 0.93, emotional function 0.97, fatigue 0.91, systemic symptoms 0.98, and worry 0.92. These statistics reflect the tool’s ability to consistently measure the health status of chronic liver disease patients, and its capacity to capture the specific health-related quality of life aspects relevant to this population [18,19].

### 2.4. Statistical Analysis

Statistical analysis was conducted using SPSS Version 23.0 and graphical representations created with GraphPad Prism. Quantitative variables were summarized using means and standard deviation or median and interquartile range, based on their distribution. Group differences based on frailty status were assessed using ANOVA for normally distributed continuous variables, Chi-square tests for categorical variables, and Fisher’s exact test for small sample sizes or infrequent categories. Correlations were analyzed using Spearman’s correlation coefficient. We used multivariable logistic regression models to determine the independent predictors associated with the chronic liver disease questionnaire (CLDQ) scores and interpreted these via odds ratios (OR) and 95% confidence intervals (CI). Statistical significance was inferred at a two-sided *p*-value of less than 0.05.

## 3. Results

### 3.1. Patient Characteristics and Selection

During the study period, a total of 294 subjects were screened for potential participation. Of these, 156 agreed to participate in the study. Several patients were excluded based on our criteria: those who had severe ascites (*n* = 7), overt hepatic encephalopathy (*n* = 5), cognitive impairment or dementia (*n* = 2), and Parkinson’s disease (*n* = 2), as well as those who did not complete the interview or physical tests (*n* = 2). After applying these exclusions, the final analysis included 138 participants.

The mean age (in years) of the patient was 47.42 ± 13.47, BMI 21.44 ± 2.89 (Kg/m^2^), MELD-Na 13.9 ± 4.67, TAMA 25.8 ± 5.46 (cm^2^), HGS 23.21± 5.71 (kg m^−2^), and gait speed 0.85 ± 0.05 (m s^−1^). The primary etiology of liver cirrhosis was alcoholism (70.2%) followed by viral infections (17.3%) and cryptogenic (8.6%). For the severity of liver cirrhosis, the mean CTP score was found to be 10.4 ± 2.25 in males and females 8.3 ± 1.60. Subsequently, 71 (51.45%) and 67 (48.55%) patients were found in the B and C categories of the CTP classification, respectively. Moreover, moderate ascites was reported by 36.2% of patients, whereas refractory ascites was reported by 25.3% (Table 1).

### 3.2. Primary Outcomes

Our study categorized a total of 138 patients into two distinct groups: Group 1, consisting of robust and pre-frail individuals, constituted 55.08% of the sample; Group 2, composed of frail individuals, made up the remaining 44.9%. To investigate the differences between these two groups, we performed a comparative statistical analysis.

Our analysis consisted of an evaluation of both continuous and categorical variables. For continuous variables, such as age, BMI, CTP score, and MELD sodium, we utilized independent *t*-tests. For categorical variables, we employed chi-square tests.

We found significant differences between the frail and non-frail groups across various parameters. The frail group, for instance, had a higher mean age in comparison to the non-frail group ((46.42 ± 13.47 years vs. 41.62 ± 9.89 years); *p*-value 0.004). Moreover, the frail group also demonstrated a substantially higher CTP score relative to the non-frail group ((11.21 ± 2.71 vs. 10.64 ± 1.67); *p*-value < 0.027). A similar pattern was seen in MELD sodium levels ((12.16 ± 3.98 vs. 10.34 ± 4.21); *p*-value < 0.028). Interestingly, when considering laboratory parameters, only sodium levels were statistically higher in the non-frail group compared to the frail group ((130.45 ± 4.92 vs. 128.65 ± 5.83); *p*-value < 0.046).

**Figure 1:**
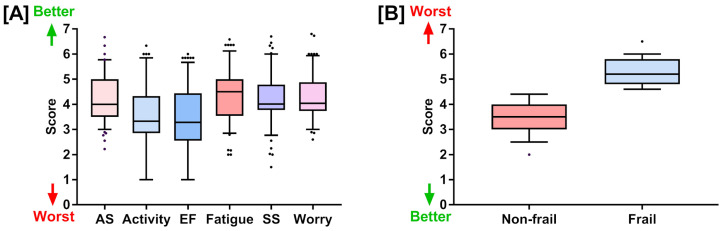
(**A**) Chronic liver disease questionnaire (CLDQ) scores for six health-related quality of life domains: abdominal symptoms, activity, emotional functions, fatigue, systemic symptoms, and worry. (**B**) Liver frailty index scores for the non-frail and frail groups. Both (**A**,**B**) are represented as box plots showing quartiles, 5th and 95th percentiles (whiskers), and minimum and maximum scores.

We also explored the mean score of six domains in the chronic liver disease questionnaire (CLDQ), which includes abdominal symptoms (4.18 ± 0.92), activity (3.45 ± 1.29), emotional function (3.37 ± 1.28), fatigue (4.38 ± 0.99), systemic symptoms (4.20 ± 1), and worry (4.28 ± 0.85). This multifaceted assessment allowed us to generate a comprehensive understanding of the patients’ lived experiences and symptoms, providing an in-depth evaluation of their health-related quality of life (Table 1 and Figure 1).

### 3.3. Secondary Outcomes

**Figure 2:**
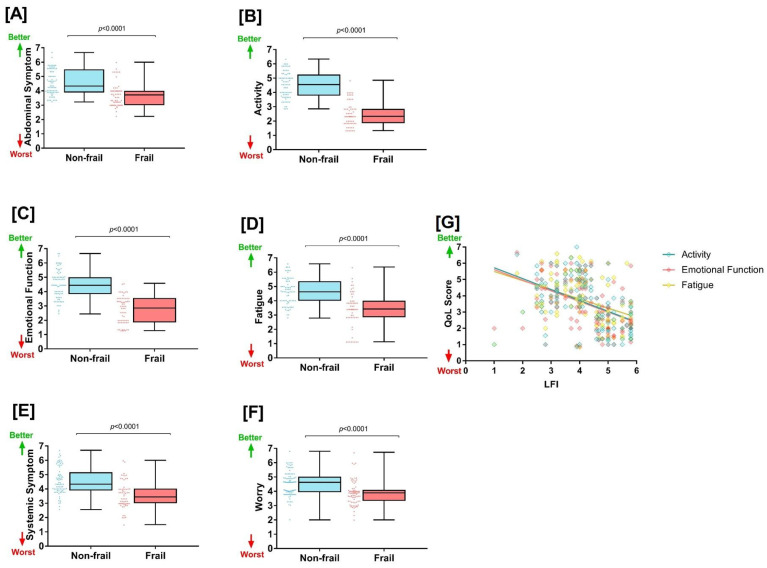
The association of liver frailty index (LFI) with chronic liver disease questionnaire (CLDQ) domains. The comparison of (**A**) abdominal symptoms, (**B**) activity, (**C**) emotional function, (**D**) fatigue, (**E**) systemic symptoms, and (**F**) worry between non-frail and frail groups are shown. (**G**) The correlation between liver frailty with CLDQ domain (activity, emotional function, and fatigue).

The CLDQ scores of all the patients in both groups are shown in Figure 2. CLDQ comprises six domains, each of which was scored on a seven-point Likert scale. For each domain, an independent *t*-test was performed to compare the mean scores between the frail and non-frail groups. Individual mean scores for non-frail and frail groups were as follows: abdominal symptoms (4.56 ± 0.85 vs. 3.73 ± 0.80), activity (4.52 ± 0.91 vs. 2.5 ± 0.85), emotional function (4.48 ± 0.99 vs. 2.72 ± 0.93), fatigue (4.68 ± 0.93 vs. 3.9 ± 1.21), systemic symptoms (4.54 ± 0.95 vs. 3.58 ± 0.94), and worry (4.58 ± 0.84 vs. 3.88 ± 0.81). Frail patients were observed to have significantly higher CLDQ scores in all six domains (*p*-value: *p* < 0.0001 for all categories) (Figure 2).

In the multivariate analysis, all six domains of the chronic liver disease questionnaire (CLDQ) were identified as associated risk factors in patients categorized as frail. Particularly, three domains—fatigue (OR 4.434 (95% CI: 3.25–7.72), *p* value = 0.002), emotional function (OR 3.58 (95% CI: 2.35–5.98), *p* value = 0.002), and activity (OR 2.53 (95% CI: 1.32–3.89), *p* value = 0.006)—showed a stronger association with frailty (Table 2).

Spearman’s rank correlation coefficient was applied to determine the correlation strength between the CLDQ domains and frailty status. A substantial correlation was observed between frailty and activity (r = −0.598, *p* = 0.001), emotional function (r = −0.511, *p* = 0.001), and fatigue (r = −0.592, *p* = 0.001) when comparing the frail group to the non-frail group.

Conversely, weaker correlations were found with abdominal symptoms (r = −0.433, *p* = 0.001), systemic symptoms (r = −0.074, *p* = 0.001), and worry (r = −0.090, *p* = 0.001). Therefore, although all CLDQ domains were associated with frailty, the strength of these associations varied (Figure 2).

## 4. Discussion

Our study, the first from the Indian subcontinent, revealed a significant prevalence of frailty among chronic liver disease (CLD) patients and its profound impact on their health-related quality of life (HRQoL), supporting previous international findings. This unique geographical context enriches the general understanding of frailty and its cultural variations [20].

We focused on a middle-aged population with liver cirrhosis, unlike traditional studies that focused on the elderly. Despite their relatively younger age, the high prevalence of frailty observed in our study population supports the notion of ‘biological’ aging that goes beyond mere chronological years. Physiological and functional impairments due to liver cirrhosis may accelerate the onset of frailty, irrespective of the patient’s actual age. Our findings echo recent literature, highlighting the relevance of frailty in the prognosis and management of patients with chronic liver disease, even in younger age groups [21]. This underscores the importance of routine screening for frailty in this patient group and opens avenues for future research on targeted interventions to reduce frailty and its associated adverse outcomes in liver cirrhosis patients of all ages [19,22].

In our study, frail patients had significantly lower sodium levels compared to non-frail patients, which is noteworthy as hyponatremia has been associated with increased frailty in patients with liver. Hyponatremia’s association with frailty may stem from multiple physiological pathways. Low serum sodium levels in cirrhotic patients can exacerbate fluid retention, leading to ascites and edema, which can further contribute to physical frailty. Additionally, hyponatremia has been linked to neurological complications, including hepatic encephalopathy, which can exacerbate cognitive impairment and precipitate frailty. Moreover, hyponatremia is reflective of the severity of liver disease and has been associated with decompensated cirrhosis, portal hypertension, and renal dysfunction, all of which can contribute to an increased risk of frailty [23]. However, while our study supports the potential of sodium as a frailty biomarker, further research is needed. The causality of the observed relationship remains unconfirmed and should be further investigated through prospective, longitudinal studies. Additionally, future studies should aim to determine optimal serum sodium cut-offs for frailty risk and examine the efficacy of sodium correction strategies in reducing frailty among patients with chronic liver disease.

Building upon our observations, it is evident that frailty in patients with chronic liver disease is a multidimensional phenomenon, encompassing not only physical limitations but also significant emotional distress. Chronic conditions, by their very nature, often impose a significant emotional burden, characterized by mood disorders such as depression, anxiety, or general emotional distress. Within our study population, the domain of ‘emotional function’ from the chronic liver disease questionnaire (CLDQ) stood out as an area of particular concern. This observation aligns with existing research demonstrating that the psychological ramifications of chronic illnesses can significantly impair patients’ perceived health status and overall quality of life. The interconnectedness of physical and emotional health is particularly relevant in the management of chronic conditions. Unaddressed emotional stress can potentially exacerbate physical symptomatology; compromise adherence to prescribed treatment regimens; and, ultimately, undermine the efficacy of therapeutic interventions. Therefore, the inclusion of psychological assessments within the healthcare paradigm for chronic diseases such as CLD is not merely beneficial but vital for comprehensive patient care [24,25].

Our study, however, is not without limitations. The sample size, initially calculated to be 385, was significantly reduced to 138, mainly due to participant dropouts and the application of exclusion criteria. This reduction might have affected the statistical power of our findings, a concern shared by similar studies investigating frailty and HRQoL in CLD patients [26].

The cross-sectional design of the study also limits our ability to establish a causal relationship between frailty and decreased HRQoL. Previous literature has also emphasized the necessity for longitudinal designs in order to elucidate these temporal relationships. The generalizability of our findings may be influenced by the sample’s geographical and demographic characteristics, with participants mainly from urban areas in Northern India. There has been an increasing call for more representative samples in health research, particularly from rural areas, given the healthcare disparities they often face [27,28,29,30].

The observed link between frailty, as measured by LFI, and decreased HRQoL suggests that the early detection and management of frailty could potentially improve the HRQoL in CLD patients. Given the role of nutrition in both liver disease progression and frailty, the impact of nutritional status and the potential benefits of nutritional interventions warrant further investigation. Nutritional therapy could play a crucial role in reducing frailty and improving HRQoL in CLD patients [31,32].

In summary, our study underscores the significance of frailty as a key factor in managing chronic liver diseases. By recognizing and understanding the impact of frailty on quality of life, clinicians can develop more comprehensive and effective care strategies for their patients with chronic liver diseases.

## 5. Conclusions

The clinical parameters of liver cirrhosis in the frail and non-frail groups were substantially different. In individuals with LC, frailty was linked to a lower CLDQ. Frailty and HRQOL are critical factors in the severity of liver cirrhosis; better control of one can reduce mortality and morbidity in patients with chronic liver cirrhosis. The potential role of CLDQ in the treatment and prevention of frailty syndrome calls for a long investigation into its clinical uses in frail or pre-frail liver cirrhotic individuals.

## Figures and Tables

**Table 1:** Demographic and clinical characteristics on the basis of frailty status for 138 patients enrolled in the study.

Characteristics	Total (N = 138)	Frail (*n* = 62, 44.92%)	Non-Frail (*n* = 76, 55.07%)	*p*-Value ^a^
Age (in years)	47.42 ± 13.47	46.32 ± 12.65	41.62 ± 9.89	0.004
Gender				
Male	118 (85.50)	60 (50.84)	58 (49.15)	0.151
Female	20 (14.49)	2 (20)	18 (90)	
Etiology				
Alcohol	97 (70.28)	40 (41.23)	57 (58.76)	
Viral	24 (17.39)	14 (58.33)	10 (41.67)	
Autoimmune	5 (3.62)	2 (40)	3 (60)	0.053
Cryptogenic	12 (8.69)	6 (50)	6 (50)	
Body mass index (Kg/m^2^)	21.44 ± 2.89	19.16 ± 1.54	20.88 ± 1.77	<0.001
Child–Turcotte–Pugh score	10.54 ±2.25	11.21 ± 2.71	10.64 ± 1.67	0.027
Child–Turcotte–Pugh classification				
B	67 (48.55)	26 (38.80)	41 (61.19)	0.212
C	71 (51.45)	36 (50.70)	35 (49.29)	
Hepatic encephalopathy				
None	96 (69.56)	36 (37.50)	60 (62.50) (55.20)	
Grade I	25 (25.36)	14 (56)	11(44)	0.064
Grade II	17 (12.32)	12 (70.58)	5 (29.41)	
MELD-Na	13.9 ± 4.67	12.16 ± 3.98	10.34 ± 4.21	0.028
Total bilirubin (mg/dL)	4.60 ± 1.81	4.35 ± 1.49	4.14 ± 0.98	0.051
Creatinine (mg/dL)	1.04 ± 0.89	0.67 ± 0.85	1.03 ± 0.59	0.372
INR	13.87 ± 2.48	11.56 ± 1.95	11.51 ± 1.88	0.148
Sodium (mEq/L)	129.47 ± 6.78	128.65 ± 5.83	130.45 ± 4.92	0.046
Ascites				
Mild	23 (16.66)	11 (47.82)	13 (56.52)	
Moderate	50 (36.23)	23 (46)	27 (54)	0.24
Refractory	35 (25.36)	19 (54.28)	16 (45.71)	
Sarcopenic parameters				
Hand grip strength (kg m^−2^)	23.21 ± 5.71	20.93 ± 3.38	22.25 ± 2.85	<0.001
Gait speed (m s^−1^)	0.85 ± 0.05	0.77 ± 0.07	0.85 ± 0.03	0.081
TAMA (cm^2^)	25.8 ± 5.46	24.68 ± 3.78	24.90 ± 3.73	0.054

Values are expressed in mean and standard deviation (Mean ± SD), and number and percentage *n* (%). MELD = model for end-stage liver disease, INR = international normalized ratio, and TAMA = total abdominal muscle area. ^a^ The *p*-value was determined by comparing the frail and non-frail groups. A *p*-value less than 0.05 was considered statistically significant.

**Table 2:** Multivariate logistic regression coefficient for the liver frailty status based on CLDQ score.

Variables	β	*p*-Value *	OR ^◊^	95% Confidence Interval		R^2^
				Lower Bound	Upper Bound	
Abdominal symptoms	−0.585	0.012	1.273	0.800	2.398	0.187
Activity	−1.618	0.006	2.539	1.325	3.893	0.597
Emotional function	−1.829	0.002	3.589	2.354	5.981	0.261
Fatigue	−1.935	0.002	4.434	3.258	7.728	0.350
Systemic symptoms	−0.815	0.023	1.843	0.858	2.760	0.015
Worry	−0.438	0.040	0.928	0.703	2.120	0.008

The reference category is: non-frail. ^◊^ OR—odds ratio. * *p*-value is significant at level 0.05.

## Data Availability

The data collected and/or evaluated in the BCAAS study are intended for academic research and can be accessed upon suitable request to the corresponding author.
